# Sensor Verification and Analytical Validation of Algorithms to Measure Gait and Balance and Pronation/Supination in Healthy Volunteers

**DOI:** 10.3390/s22166275

**Published:** 2022-08-20

**Authors:** Robert Ellis, Peter Kelly, Chengrui Huang, Andrew Pearlmutter, Elena S. Izmailova

**Affiliations:** Koneksa Health, New York, NY 10038, USA

**Keywords:** technical verification, analytical validation, accelerometer, gait and balance, walking, pronation, Parkinson’s disease

## Abstract

Numerous studies have sought to demonstrate the utility of digital measures of motor function in Parkinson’s disease. Frameworks, such as V3, document digital measure development: technical verification, analytical and clinical validation. We present the results of a study to (1) technically verify accelerometers in an Apple iPhone 8 Plus and ActiGraph GT9X versus an oscillating table and (2) analytically validate software tasks for walking and pronation/supination on the iPhone plus passively detect walking measures with the ActiGraph in healthy volunteers versus human raters. In technical verification, 99.4% of iPhone and 91% of ActiGraph tests show good or excellent agreement versus the oscillating table as the gold standard. For the iPhone software task and algorithms, intraclass correlation coefficients (ICCs) > 0.75 are achieved versus the human raters for measures when walking distance is >10 s and pronation/supination when the arm is rotated more than two times. Passively detected walking start and end time was accurate to approx. 1 s and walking measures were accurate to one unit, e.g., one step. The results suggest that the Apple iPhone and ActiGraph GT9X accelerometers are fit for purpose and that task and passively collected measures are sufficiently analytically valid to assess usability and clinical validity in Parkinson’s patients.

## 1. Introduction

Parkinson’s disease (PD) is a neurological disorder characterized by a progressive worsening of motor function, resulting in impacts to speech, gait, balance, limb dexterity, tremor and other symptoms. The disease is heterogenous and variation in signs, symptoms and impacts can be observed between patients [1]. The gold standard for assessing the efficacy of novel therapies in Parkinson’s disease is the MDS-UPDRS [2], specifically Parts II and III which assess non-motor and motor impacts of disease on daily living. In recent years, there has been significant interest in the development of other tools to measure the symptoms of Parkinson’s disease which leverage digital health technologies (DHT).

Multiple advances have been made in the development of sensor-based measures which use software to measure motor fluctuations in patients with Parkinson’s disease via sensors such as accelerometers and gyroscopes and then interpret that motion using algorithms. Such sensors can be implemented passively, i.e., recording motion during daily activities without patient input, or actively, by asking the patient to complete an instructed task implemented via software. Examples include the pioneering work of the mPower study [3], Lipsmeier et al. (2018) [4] and more recently by Burq et al. (2022) [5]. A recent literature review by Barrachina-Fernandez et al. (2021) [6] presents a summary of 10 other studies in which machine learning techniques are used to measure motor fluctuations in patients with Parkinson’s disease. In general terms, the aforementioned studies examine whether the measures generated are able to measure changes due to medication or disease and/or demonstrate construct validity with the MDS-UPDRS (Table 1). While the majority of papers demonstrate the ability to measure motor fluctuations, the work undertaken is not presented within the context of a development framework, such as V3 [7], that iteratively establishes the evidence of (1) clinical association, (2) technical verification, (3) analytical validity and (4) clinical validity. It is important to note that an evidentiary package must contain data to support each step described in relevant frameworks to render a sensor with companion data processing algorithms to be appropriate for use in clinical trials.

The measurement of new therapies’ efficacy using DHT which leverage sensors requires that such tools be independently validated and appropriate for the context of use. In recent years, we have witnessed substantial development of device validation frameworks by both regulatory agencies and the scientific community. The Food and Drug Administration has published two guidance documents: “Software as a Medical Device (SAMD)” [8] and “Patient Reported Outcome Measures” (PRO Guidance) [9]. However, not all DHT-based solutions are intended to be used as a medical device and not all DHT measures are intended to measure patient outcomes. While the aforementioned guidance documents detail many of the steps required to establish utility and validity for a given intended use, an overall framework was lacking. This gap and the growing experimentation and interest in the use of DHT in clinical research prompted the scientific community to sponsor the V3 framework, where V3 stands for Verification, analytical Validation and clinical Validation [7]. More recently the Food and Drug Administration published specific draft guidance on the use of DHT for remote data acquisition in clinical investigations [10]. We note that more recent frameworks such as V3 [7] and the DHT guidance [10] do not require the determination of clinical association and that this requirement is defined in the SAMD guidance [8].

The work presented in this paper covers the technical verification and analytical validation of measures recorded passively and via two software instructed tasks of three symptoms relevant to Parkinson’s disease: gait, balance and pronation/supination. The measures were selected to align to signs and symptoms identified in concept development from the combination of a literature review, clinical expert input and PD patient engagement [11]. The work presented in this paper preceded result-dependent studies in patients with Parkinson’s disease to assess usability and clinical validity [12]. This work is a preliminary step that needs to be completed prior to testing the technology and data processing algorithms in patients with PD. First time testing of novel technologies with unknown performance may require multiple iterations and present a burden to patients.

The two devices used in the work are an Apple iPhone 8 Plus (iPhone) and an ActiGraph GT9X (ActiGraph). The ActiGraph was used to passively capture walking motion during the day and the iPhone, plus accompanying software, was used for point in time assessment to capture motion as subjects followed instructions provided by a mobile app. Technical verification was performed using the bench test evaluation of the device accelerometers and analytical validation was performed in healthy adult volunteers under controlled conditions—two distinct parts of the same observational study.

## 2. Materials and Methods

### 2.1. Study Design

#### 2.1.1. Study Setup

The study was conducted as a single-center observational study enrolling healthy volunteer employees from Koneksa Health for the assessment of analytical validity. The inclusion criteria included participants aged between 18 and 65 and able to walk without the use of a walking aid. Exclusion criteria included any self-reported diagnosis of PD, other ambulation limitations or taking any medication to improve mobility or relieve pain during normal activities of daily living. The study was conducted in accordance with the Declaration of Helsinki and approved by the Institutional Review Board of Solutions IRB (Arizona, TX, USA).

Participants were enrolled according to study inclusion/exclusion criteria and provided written consent. We define a task as the software installed on the iPhone and a test as the execution of one task by one participant with a given configuration. All tests were executed in a space secured for the purpose of the study, free of obstacles and approx. 20 m in length. Execution of study tests was completed by 3 trained raters. One rater performed the role of test supervisor and primary timekeeper and the other two raters either acted as additional timekeepers or counted/measured the required measures.

Participants were trained at study enrollment in the requirements for each task and test. All walking tests were conducted via a marked course. Participants completed each of the iPhone application and ActiGraph walking tests and repeated them 4 times, split over 2 site visits. A test log was used to record subject ID, visit, task, test identifier, test repeat number, start and end time and then rater-recorded measures. At the end of each visit, the test log was reviewed by an independent team member and any quality control issues were flagged for follow up. Any corrections made as a result of issues flagged were logged.

For both the iPhone tasks, two types of tests were conducted: analytical validation and operational tolerance. In the analytical validation tests, the participant was instructed to perform the test for a specific duration of time from less than or equal to the default configuration of the test. In the operational tolerance tests, the participant was instructed to complete the test with a predefined variation from default instructions. Operational tolerance tests were conducted because in final form the system was intended to be used unsupervised by patients with Parkinson’s disease at home when deviations from an instructed execution may occur. It was therefore necessary to determine whether the algorithm output was tolerant of deviations.

#### 2.1.2. Sample Size Estimation

The sample size calculation objective is to achieve an interval estimate that has sufficient precision. Increasing the sample size (the number of subjects) and/or the number of repeated measures per subject can increase the precision, i.e., decrease the width of the 95% confidence interval (CI) for agreement as measured by the intraclass correlation coefficient (ICC). For a precise and statistically meaningful outcome for precision, the following must be true:

- Anticipated ICC > 0.75 (good agreement);
- Anticipated ICC plus half width of 95th percentile CI ≤ 1;
- Anticipated ICC minus half width of 95th percentile CI ≥ 0.75.

Equation # 7, established by Shoukri et al. (2014) [13], was used to determine the number of repeat tests given inputs of a sample size of 10 participants, anticipated ICC and number of repeat measures. The resulting width, lower and upper bounds of the 95th percentile CI for ICC were calculated. Calculations determined that:

- Three or four repeats are required for the ICC upper bound to be less than one;
- To retain the lower bound, more than 4 repeats are required if the ICC is less than 0.9.

Absent example studies, empirical determination of anticipated ICC cannot be estimated. As a result, the estimate of test–retest reliability for the MDS-UPDRS Part III determined by Siderowf et al. (2002) [14] of 0.9 with a lower bound of 0.8 is used. This threshold sets the target anticipated ICC for the analytical validation of objective measures of PD symptoms to be the same as their MDS-UPDRS Part III counterparts. Equation #7 determines that 4 repeat tests are required per subject to achieve an anticipated ICC of 0.9.

### 2.2. Materials

#### 2.2.1. Hardware

This study used 2 commercial off the shelf devices from third party manufacturers: an Apple iPhone 8 Plus^®^, Cupertino, CA, USA, (iPhone) with an accelerometer and gyroscope [15] and ActiGraph GT9X Link^®^, Pensacola FL, USA, (ActiGraph), with an accelerometer [16]. The iPhone 8 Plus is CE marked. The ActiGraph device is a class II 510(k) exempt exercise measuring device under application number K080545. The ActiGraph was configured with sleep mode enabled. This setting is routine for the device and pauses the recording of high frequency data when no/low levels of motion are detected.

**Figure 1:**
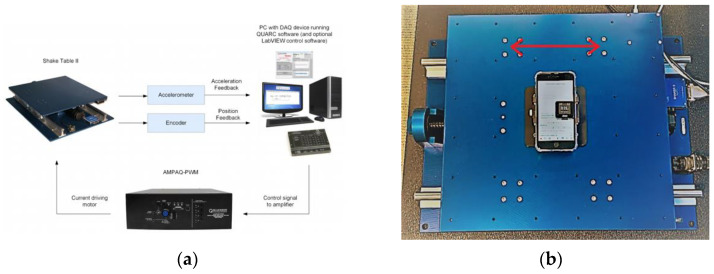
Quanser Shake Table II system; (**a**) system components; (**b**) iPhone and ActiGraph mounted in a custom cradle bolted to the moving table.

Technical verification of device accelerometer accuracy and precision was conducted using a Quanser Shake Table II^®^ [17], Ontario Canada, (Shake Table) and accompanying QUARC software [18], shown in Figure 1. The iPhone gyroscope was not technically verified on the basis of other published testing results [19]. The Shake Table is an oscillating table which contains an onboard accelerometer and can be configured to move back and forth over a range of amplitudes and frequencies. A custom cradle was manufactured by Quanser to securely mount the iPhone and ActiGraph devices to the table. The QUARC software test configurations were developed in MathWorks MATLAB^®^ R2019b (Natick, MA, USA) [20].

Raw accelerometer data recorded on the iPhone was retrieved using Bernd Thomas’ SensorLog application v3.0 (Stuttgart, Germany) [21]. The raw data recorded on the ActiGraph were retrieved using ActiGraph corporation’s CentrePoint platform and accompanying ActiSync software (ActiGraph, Pensacola, FL, USA) [22,23]. The data from the Shake Table and all devices were down sampled to 30Hz as the lowest common frequency. This results in a dataset of 300 samples per amplitude and frequency test configuration.

#### 2.2.2. Software

The study evaluated the reliability, accuracy and precision of an application (v1.2.78) designed and built by Koneksa Health, New York, NY, USA and installed on an iPhone with iOS12 or more recent operating system. The software application implemented Apple ResearchKit v1.5 (Apple, Cupertino, CA, USA) to access iPhone sensor data and included 2 task-based assessments which instruct the participant to first complete a 20 s walk to assess gait and balance, and second, to hold the phone arm outstretched and rotate the arm for 20 s to assess pronation/supination.

The ActiGraph device was worn on the dominant wrist and passively collected body motion while the participant was walking as instructed by the study staff. The software installed on the iPhone is paired with the participant through a one-time pairing code and transmits recorded sensor data and other task metadata to “Koneksa Compare”, a Software as a Service (SaaS) platform.

#### 2.2.3. Algorithms

**Figure 2:**
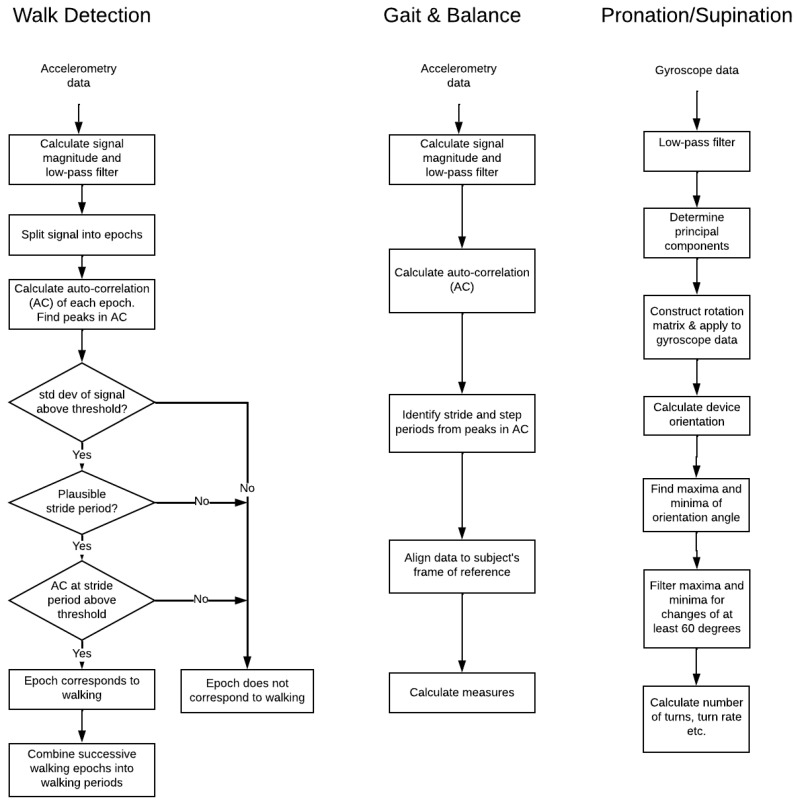
Flow chart of the data processing algorithms for walk detection, gait and balance and pronation/supination.

Three algorithms were evaluated in this study: (1) walk detection, (2) gait and balance and (3) pronation/supination. Motion such as walking or arm rotation results in repetitive patterns in sensor raw data which can be defined and then extracted from the raw data using standard signal processing techniques. These techniques include the application of filters to remove noise. The walk detection and gait and balance algorithms were applied to accelerometer data recorded via the ActiGraph, the gait and balance algorithm to the iPhone walk test and the pronation/supination algorithm applied to the gyroscope data recorded for the pronation/supination task. Version 1.2 of the algorithm was implemented with v1.2.78 of the mobile application. A summary of the algorithms is documented in Table 2 and Figure 2.

The algorithms were initially developed in MATLAB R2019b and then double programmed in Python 3.6.5 (Fredericksburg, VA, USA) [24] from a documented step-by-step mathematical definition. The gait and balance algorithm was developed leveraging the data recorded in the mPower study [3]. The walk detection and pronation/supination algorithms were developed through iterative prototypes. The double programming agreement threshold was set to 1.0^−6^—i.e., the output of both implementations in MATLAB and Python must agree for the same input data to be better than one in a million. The Python implementation of the algorithms was released as v1.2 and used to process data from the iPhone software and ActiGraph in this study.

**Figure 3:**
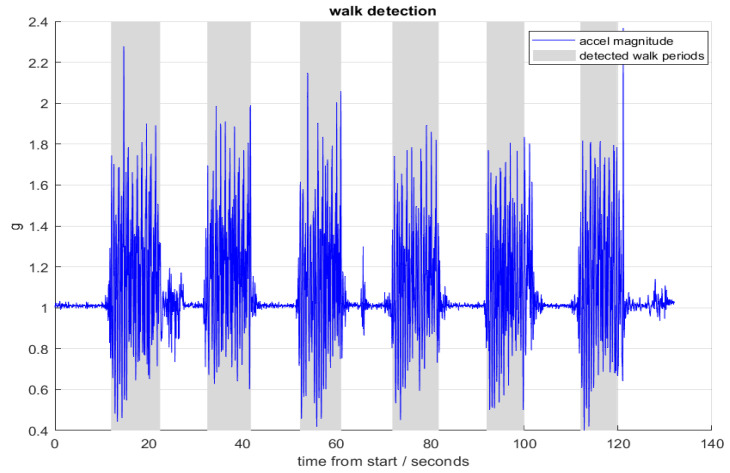
An example of raw acceleration data from ActiGraph and the detected walking periods derived from it using the walk detection algorithm. In this example, the subject was alternately walking for 10 s then stopping for 10 s, repeated 6 times.

**Figure 4:**
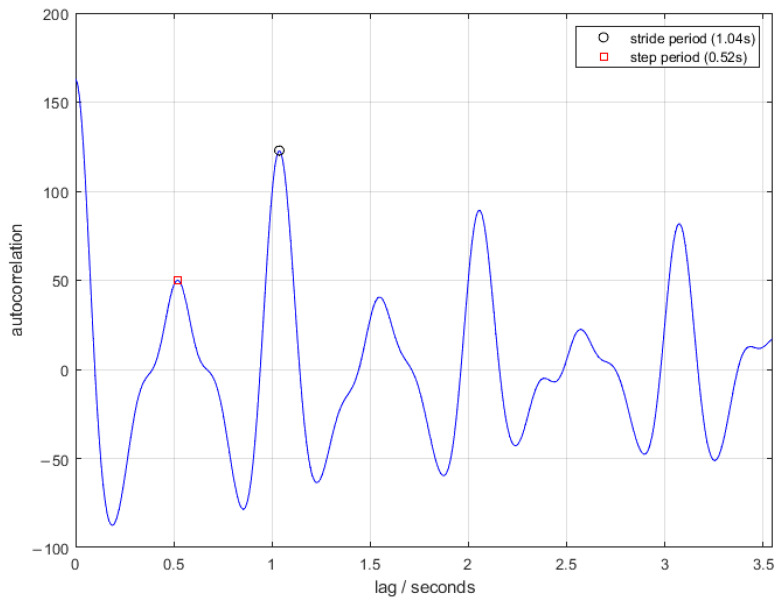
An example of the autocorrelation calculated from iPhone acceleration data in the gait and balance algorithm. The peaks in the autocorrelation corresponding to the stride and step periods, as calculated by the algorithm, are indicated.

**Figure 5:**
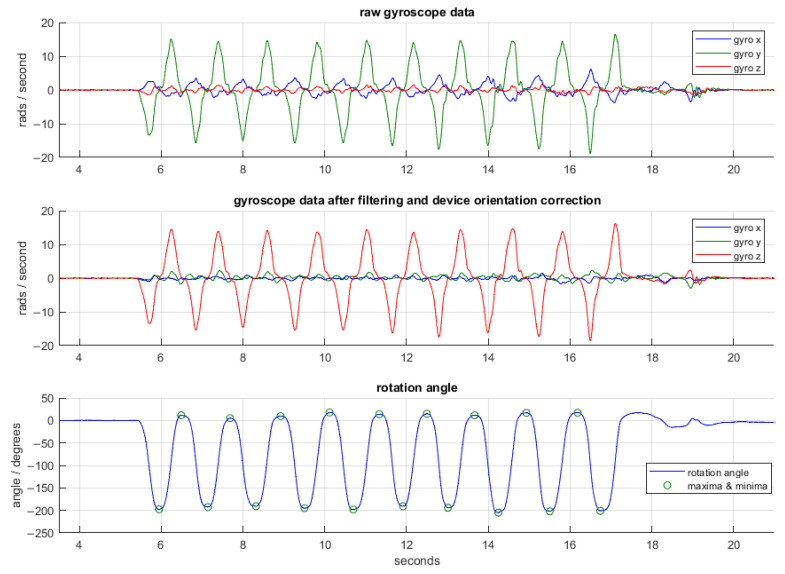
An example of the pronation/supination algorithm. Top plot shows the raw triaxial gyroscope data from an iPhone. Middle plot is after low pass filtering and correction for device orientation, such that the rotational motion is primarily around the z axis. Bottom plot indicates the rotation angle and its maxima and minima; each maxima/minima pair represents one turn.

The walk detection and then gait and balance algorithm were applied to the ActiGraph accelerometer data collected in the passive walking test. The gait and balance algorithm was applied to the accelerometer data recorded via the iPhone walking task. The pronation/supination algorithm was applied to the iPhone pronation/supination task. The measures examined in the study are documented by the algorithm in Table 3. Figure 3, Figure 4 and Figure 5 show examples of the algorithms applied to data collected in this study.

#### 2.2.4. Statistical Analysis

Statistical analysis of both the output of technical and analytical validation data was conducted using R Foundation’s R software v3.5.1 (Vienna, Austria).

For the iPhone walk and pronation/supination tests, the intraclass correlation coefficient (ICC) was used to determine agreement. A single rating, absolute agreement was calculated using a two-way mixed effects calculation. ICC was chosen because it is an appropriate test for agreement between numerical values in 2 or more sets of data [25]. ICC output was categorized according to Koo et al. (2017) [26], i.e., ICC above 0.9 is interpreted as excellent, between 0.75 and 0.9 as good, between 0.5 and 0.75 as moderate and below 0.5 as poor.

For the ActiGraph passive walk detection assessment of gait measure, analytical validity is first dependent on demonstrating the analytical validity of the algorithm versus rater start and end time for walk periods. Assessing analytical validity using ICC is not appropriate for the start and end time of a walking period because the differences between rater start times for each period are zero and therefore the ICC is zero. For the ActiGraph passive measures, analytical validity was assessed via mean absolute error (MAE), root mean square error (RMSE) and mean absolute percentage Error (MAPE), between algorithm and rater measures.

### 2.3. Technical Verification

The Shake Table was installed and operationally qualified according to the manufacturer’s instructions and built-in calibration software [27]. The iPhone and ActiGraph devices were concurrently mounted on the Shake Table and subject to increasing amplitude of oscillation between 5 and 60 mm and frequencies of oscillation between 0.5 and 10 Hz.

The Shake Table was operated by 2 operators (A and B) trained in the hardware and software. A single test is defined as one specific combination of amplitude and frequency. Each test oscillated the table for 10 s and the Shake Table was stationary for a period of 15 s between tests. Each test was repeated 4 times with operator A conducting the test and operator B verifying the accompanying test log in 2 of the 4 tests and then operators swapping roles for the other 2 of 4 tests. A total of 42 amplitude and frequency configurations were tested and each repeated 4 times. A test log was completed with the configuration of each test run, operator roles plus start and end of test timestamps. All sensor data were downloaded from respective devices and export file names logged in the test log against tests covered. The output of the Shake Table onboard accelerometer was also exported, and file names stored with the test log.

The test log was then used to identify each 10 s test within each raw data file. Nominal peak acceleration (a), measured in units of g, was calculated using the standard formula a =$\;{\left(2\pi f\right)}^{2}Ag$ where A is amplitude in mm, f frequency in Hz and g is the acceleration due to gravity at 9860 mm/s^2^.

The ICC for agreement was calculated between the Shake Table accelerometer (gold standard) and each device with results tabulated and plotted by device. The Koo et al. (2016) [26] ICC categorization was then applied to each calculated ICC.

### 2.4. Analytical Validation

#### 2.4.1. iPhone Walking Task

The default configuration of the 20 s walk instructs the participant to put the iPhone in a tight pocket, a countdown period of 5 s is observed and then the participant is instructed by the software voice prompt to walk for 20 s, turning if necessary and finally to stop walking and then submit the test results. The task also the placement of the iPhone in a shoulder bag. The submission of task data completes the task.

The configuration for all tests conducted is shown in Table 4 and covers both tests for analytical validity and operational tolerance. For each test, one rater recorded the start and end time of the test and the other two raters counted the number of steps walked by the participant and the total distance walked with a measuring tape. The participant was allowed to turn around and continue walking if they reached the end of the marked course.

#### 2.4.2. iPhone Pronation/Supination Task

The default configuration of the pronation/supination task instructs the participant to start the task then hold their arm outstretched with the phone screen palm up using the dominant arm. The participant then rotates the phone palm up and palm down as many times as possible within a duration of 20 s. At the end of the test, the participant places the phone back down on a table and submits the task. Submitting the task completes the test.

The configuration for all tests conducted is shown in Table 5 and covers tests for both the determination of analytical validity and operational tolerance. For each test, one rater recorded the start and end time for the test and the other two raters counted the number of complete turns of the iPhone. The first test was supervised (rater guides the participant through the test) and the subsequent tests were unsupervised (participant left to review task instructions in the application as needed).

#### 2.4.3. ActiGraph Passive Walking Detection and Gait

This testing focused on whether the walk detection algorithm could accurately and reliably identify periods of walking from other body motion recorded. The data are collected passively, i.e., there is no software instructing the participant when to walk as implemented in the iPhone walking task.

The ActiGraph device is intended to be worn during waking hours and then raw accelerometer data for the worn duration is processed to extract walking periods. For this test a number of predefined walk durations were examined. Walk detected periods were then processed by the gait and balance algorithm to extract walking measures. Table 6 documents the different test configurations examined.

### 2.5. Data Processing

All iPhone task raw data with associated task, device and subject identifiers plus timestamps are stored centrally by the SaaS platform. The same platform associates subject to iPhone, application installation and ActiGraph device identifiers. The ActiGraph data were downloaded from the ActiGraph CentrePoint platform.

Rater test logs were reviewed by two independent reviewers, approved then stored for later processing. The gait and balance and pronation/supination algorithms were applied to each of their respective iPhone test datasets and combined into a single rater/algorithm analysis dataset by subject, visit, task, test, test repeat and then rater and algorithm measures. Rater-derived measures were calculated where needed for measuring parity. For example, rater average gait speed was calculated by dividing the rater-recorded distance by the rater-recorded test duration. The other rater-derived measure was rater stride period which was calculated by dividing the rater-recorded duration by half the number of rater-recorded steps.

The walk detection and gait and balance algorithms were applied to the ActiGraph raw data as downloaded from CentrePoint. A combined rater/algorithm analysis dataset was produced by aligning the rater-logged overall start and end time of the test by subject, visit, test and test repeat.

## 3. Results

### 3.1. Technical Verification

**Figure 6:**
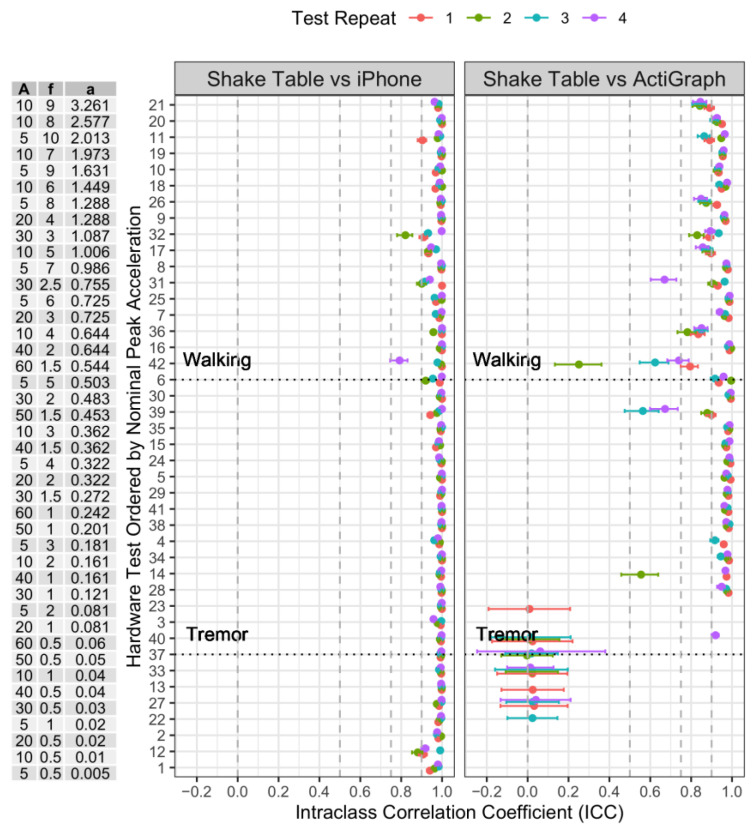
Amplitude (A) and frequency (f) of test configuration with calculated nominal peak acceleration (a) against ICC for agreement by device with the Shake Table—ordered in descending order of nominal peak acceleration. To aid in interpretation, the estimates of acceleration observed in walking calculated from the mPower study dataset (~0.5 g) [3] and for tremor (~0.05 g) [28,29] are overlaid. Each of the 4 test repeats is plotted on each chart.

Results were analyzed using a device. Figure 6 shows the results for all tests for both devices. The results for both devices are summarized in Table 7 where an ICC of 0.75 is used as a cut off to align with Koo et al. (2017) [26] rating of good or excellent. The majority of iPhone ICCs are greater than 0.75. There is a marked drop in agreement for the ActiGraph device below 0.1 g, which is the threshold for sleep idle mode.

### 3.2. Analytical Validation

#### 3.2.1. iPhone Walking Task

All results for the analytical validity tests where the participant walked for 10 s or longer demonstrate ICC > 0.75, i.e., good or excellent agreement, for measures other than duration (Table 8). The ICCs for the fixed duration tests are poor. This is expected because the standard deviation for rater duration is close to zero given that the duration is the independent variable. The ICC for operational tolerance tests, i.e., placing the iPhone in a loose pocket or carrying it in a shoulder bag, are comparable to the analytical validation tests where the iPhone is placed in a tight trouser pocket.

#### 3.2.2. iPhone Pronation/Supination Task

Table 9 shows the results for ICC by test type and measure. All but one ICC are >0.75, i.e., good or excellent. The exception is the count of turns for the analytical validation test. The ICC for the count of turns for the operational tolerance tests is greater than the ICC for analytical validity. The ICC for count of turns is lower for the analytical validation test than the operational tolerance test. The ICC for rotation rate is lowest for the operational tolerance test when the phone is only turned twice.

#### 3.2.3. ActiGraph Passive Walk Detection and Gait Measures

Table 10 shows the MAE, RMSE and MAPE for passive walk detection, by the start and end time of detected walk, then rater-aligned measures. The results for the 10 s and 20 s walk are comparable. Errors in start and end time are small, compared to the duration of the walk test. Errors in step count are less than or approximately equal to one step. Errors in stride period are less than one second.

## 4. Discussion

This study examined the technical verification of the ActiGraph and iPhone accelerometers and then the analytical validity of software and algorithms to measure gait and balance as well as pronation/supination in healthy volunteers under controlled conditions. The main objective of the work was to demonstrate that the device accelerometers are sufficiently accurate, precise and reliable over the range of accelerations of interest and that the software and algorithms warrant the assessment of usability and clinical validation in patients with Parkinson’s disease. Completing technical and analytical validation prior to clinical validation allows the determination of device and measure validity prior to establishing whether the same devices and measures can capture symptoms of disease or treatment effect.

The results for technical verification suggest that the iPhone and ActiGraph devices have sufficient accuracy, precision and reliability to capture walking motion. There are some notable outliers such as the technical validity of the ActiGraph device at accelerations less than 0.1 g, calculation of gait measures for walking periods less than 10 s and turn count for pronation/supination when completing the assessment as instructed. Our sensor verification work demonstrates the need to follow the steps for assessing technology technical performance, as stipulated in the V3 framework [7] and the FDA regulatory guidance document [10]. Device clearance by US regulators is intended for healthcare delivery and does not render a device of interest to be appropriate for use in clinical trials. As indicated by our results, the ActiGraph accelerometer performance is not uniform across tested accelerations. Our results for this device are in line with the intended use as a medical device: sleep and physical activity in the general population, highlighting the need for sensor verification irrespective of regulatory clearance of a device of interest [10]. For example, the assessment of postural tremor, an important feature of PD, can be also carried out by means of an accelerometer. This assessment was out of the scope of the experiment described in this manuscript, as the analytical validation was limited to normal healthy volunteers who do not have an appreciable tremor. The frequencies and accelerations are determined for PD [30]. Our data indicate that the ActiGraph device may be less suitable for tremor detection when the sleep idle mode is enabled compared with the iPhone. When sleep idle mode is enabled, this device only collects data when the acceleration level exceeds certain thresholds, to lengthen battery life and optimize memory use.

The solution described in this manuscript allows researchers to extract features of gait and balance from an iPhone or a wrist worn device. We believe that the use of these devices along with body placement location is more convenient for patients and may result in higher data generation compliance in patients with disease. Gait and balance characteristics generated by means of body worn sensors and associated algorithms are often extracted from sensors attached to various locations on the body, such as shanks, spine, head, pelvis and feet [31]. A systematic review of validity and reliability of consumer-wearable fitness trackers suggests higher accuracy for a tracker when placed on a hip [32]. However, our study indicates that it is possible to achieve an accurate detection of step count, walking distance, stride period and walking speed with a wrist-worn device. Moreover, we demonstrated that the data processing algorithms are operationally tolerant to deviations expected under free life conditions. We acknowledge that in other studies [5,33,34], a wrist-worn device has also been used to capture motor function fluctuations, such as dyskinesia and resting tremor, over using a smartphone such as the iPhone.

There are many examples of sensor- and software-based tools which measure the motor symptoms of Parkinson’s disease [3,4,5,30]. To our knowledge, this study may be one of the first studies that describes the stepwise process of verification and analytical validation of measures to assess motor function prior to testing in Parkinson’s disease patients and following a formal framework to establish fitness for purpose, such as we now know as V3 [7]. Our results suggest that the iPhone application software and algorithms are sufficiently valid in healthy subjects, against an ICC threshold of 0.75 and test–retest reliability for the MDS-UPDRS Part III determined by Siderowf et al. (2002) [14] of 0.9. The results compliment the growing body of work in this field such as that of Lipsmeier et al. (2018) [4] and Burq et al. (2021) [5] which established elements of usability and clinical validation (Table 1).

The results for the analytical validity of passive walk detection and gait measures recorded via the wrist-worn ActiGraph are all within or close to the unit measurement for walking periods longer than 10 s. By unit measurement we mean one second of walk start or stop, one step or one stride. To provide context for the results, Tedesco et al. (2019) [35] report MAPE step data for six devices (e.g., ActiGraph GT9X, Philips Health Watch and Garmin Vivosmart) worn in different positions (e.g., wrist, ankle) and the lowest reported MAPE for wrist-worn ActiGraph is 62.99%. Our results show smaller errors of measurements for step count, walking speed and distance. We tested activities related to walking and hand movements only; our experiments did not include traveling up and down stairs, and we did not include any unstructured activities—this altogether could account for different error rates.

Our study has several limitations. This is a small, single-center study with a limited number of study subjects, who were healthy volunteers. This study population was chosen intentionally to assess the performance of the selected algorithms in conjunction with selected sensors prior to testing in PD patients. Another limitation of the study is that the analytical validation of algorithm output was restricted to measures which can be observed or measured by human raters. The human rater comparison approach is common and was used in the study that received a positive opinion from the European Medicines Agency for use in clinical trials [36] to quantify patients’ ambulation abilities in Duchenne muscular dystrophy.

Our study also suggests opportunities for further research and improvements to algorithms, notably repeating sensor verification of ActiGraph with sleep idle mode disabled and future models of the iPhone. The next step is clinical validation in patients with Parkinson’s disease which may require algorithm adjustments. However, the work presented in the paper provides a foundational normative study to enable future work in disease conditions.

## 5. Conclusions

In this study, we presented the activities conducted to determine the technical performance of selected device accelerometers and then the analytical validation of a combination of software and algorithms to calculate the measures of motion in healthy individuals. We intentionally focused human subject testing on healthy individuals so that we could determine the analytical validity of the algorithm independent of disease. This is an example of systematic, stepwise evaluation of body worn sensors with data processing algorithms prior to testing in patients with disease.

Technical verification of accelerometer accuracy was conducted by comparing sensor output from both an iPhone and ActiGraph GT9X versus a Quanser Shake Table II. The accelerometers of both devices demonstrated good or excellent agreement, as measured by the intraclass correlation coefficient, for the intended use of capturing body motion.

Having verified sensor performance, the analytical validation of point in time task-based measures and passively detected walking was completed via a number of supervised and unsupervised tasks, with algorithm-generated measures compared to aligned measures compiled by trained human raters. The agreement with human raters ranged from good to excellent, suggesting future testing of the above-described technology for use in clinical trials.

Overall, the devices, sensors, software and algorithms are sufficiently fit for purpose to proceed with the examination of usability and clinical validity in patients with Parkinson’s disease. These activities are ongoing at this time.

## 6. Patents

U.S. patent (number US11307050B2) “Method and device for high accuracy measurements of steps and strides agnostic to wear position”.

## Figures and Tables

**Table 1:** A brief literature summary to highlight the main findings of the studies referenced which evaluate wearable technologies that assess the features of Parkinson’s disease. Development framework alignment describes published evidence alignment to frameworks such as V3 or otherwise available at the time of publication.

Study	Study Objectives	Key Findings	Development Framework Alignment
Bot et al., 2016. The mPower study, Parkinson disease mobile data collected using ResearchKit [3].	An observational smartphone-based study to evaluate the feasibility of remotely collecting frequent information about the daily changes in symptom severity and their sensitivity to medication in PD.	Established a database of sensor data collected in PD patients plus candidate disease features for several tasks including memory, finger tap, voice and walking. Data subsequently hosted for other approved researchers to access.	The data were derived from Apple iPhone devices with proprietary technical validation. Frameworks available at the time were not leveraged in the research.
Lipsmeier et al., 2018. Evaluation of smartphone-based testing to generate exploratory outcome measures in a phase 1 Parkinson’s disease clinical trial [4].	The study assessed the feasibility, reliability and clinical validity of smartphone-based digital biomarkers of PD in a clinical trial setting.	Acceptable adherence among study participants. Sensor-based features showed moderate-to-excellent test–retest reliability (average ICC 0.84). All active test (sustained phonation, rest tremor, postural tremor, finger-tapping, balance and gait) features, except sustained phonation, were significantly related to corresponding MDS-UPRDS clinical severity ratings.	Sensor verification was not published. Analytical validation of accuracy of data processing algorithms was not established. The study’s main focus was clinical validation to compare sensor-based features with MDS-UPDRS in subjects with PD and healthy controls.
Barrachina-Fernandez et al., 2021 Wearable technology to detect motor fluctuations in Parkinson’s disease patients: current state and challenges [6].	A systematic review of the utilization of sensors for identifying motor fluctuations in PD patients (on and off states) and the application of machine learning techniques.	The study highlighted that the two most influential factors in the good performance of the classification problem are the type of features utilized and the type of model.	The studies selected for review did not follow technology evaluation according to frameworks required for assessing technology use in clinical trials. The authors do not consider technology evaluation or analytical validation of measures as a condition of inclusion in the analysis.
Burq, M. et al. (2022) Virtual exam for Parkinson’s disease enables frequent and reliable remote measurements of motor function [5].	Clinical evaluation of smartwatch-based active assessment that enables unsupervised measurement of motor signs of PD.	The study established patient engagement, usability in addition to comparing the smartwatch-based modern features with MDS-UPDRS scale items.	Sensor verification and analytical validation of data processing algorithms were not established.
Sensor verification and analytical validation of algorithms to measure gait and balance and pronation/supination in healthy volunteers [current manuscript].	Technical verification of accelerometers in an Apple iPhone 8 Plus and ActiGraph GT9X versus an oscillating table; analytical validation of software tasks for walking and pronation/supination in healthy volunteers versus human raters.	The study followed the V3 framework and ascertained that selected sensors and algorithms processing accelerometry data are accurate and appropriate to use in clinical validation studies in patients with Parkinson’s disease.	This study followed the framework and FDA guidance on DHT use for remote data collection in clinical investigations. This is a preliminary step to ascertain technology performance prior to testing in patients.

**Table 2:** Algorithm summary.

Algorithm	Measure
Walk Detection	Calculate the magnitude of the acceleration vector $r=\sqrt{x^{2}+y^{2}+z^{2}}$ where *x*, *y* and *z* are the three orthogonal components of the acceleration vector. Remove high-frequency signal not related to walking by applying a low-pass filter with cut-off frequency of approximately 10 Hz. Split signal into 10 s overlapping epochs. Apply a Hamming window to the data from each epoch. Calculate the autocorrelation of each epoch. Identify the peaks in the autocorrelation signal (Peaks correspond to periodicities within the signal). Apply walking signal thresholds to each epoch. The standard deviation of the signal should be above a given threshold (since walking is a vigorous activity). The repeat period of the signal should correspond to a plausible stride period. Autocorrelation at the stride period should be above a given threshold, i.e., the signal must be repetitive. Extract epochs which score as walking. Connect consecutive walking epochs into a single walking period. Calculate start and end time of each walking period.
Gait and Balance	Calculate the magnitude of the acceleration vector. Remove high-frequency signal not related to walking by applying a low-pass filter with cut-off frequency of approximately 10 Hz. Calculate the autocorrelation of the signal. Identify peaks in autocorrelation signal. Identify stride and step periods from peaks. Align device and patient orientation by performing principal component analysis to identify the vertical direction; the direction of walking; and the side-to-side direction. Calculate measures.
Pronation/Supination	Remove high-frequency signal not related to rotation from the gyroscope raw data by applying a low-pass filter with cut-off frequency of approximately 20 Hz. Correct device orientation by performing principal component analysis to determine the axis of rotation. Integrate the angular velocity around the axis of rotation to determine rotation angle as a function of time. Identify turns in the axis of rotation. To be classified as a turn the orientation must change by at least 60 degrees. Calculate measures.

**Table 3:** Algorithm measures examined in this study.

Algorithm	Measure
Walk Detection	Start time of detected walking period (Unix timestamp) End time of detected walking period (Unix timestamp)
Gait and Balance	Duration of walk (s) Number of steps (count) Distance walked (m) Average walking speed (m/s) Average stride period (s)
Pronation/Supination	Number of completed turns (count) Average rotation rate (turns/s)

**Table 4:** iPhone walking task test configurations.

Test Type	Test Configuration
Analytical Validity	Stand still for 10 s; Start the task; Place the phone in a trouser pocket; Follow voice prompts to start walking; Stop walking when instructed by the study team; Stop and stand still for 10 s; This test configuration was completed for walks of duration 5, 10, 15 and 20 s.
Operational Tolerance	Stand still for 10 s; Start the task; Walk for 20 s as instructed by the task; Follow voice prompts to start walking; Stop and stand still for 10s. This test configuration was completed with the iPhone placed in a loose trouser pocket, typical of sports shorts, and in a shoulder bag.

**Table 5:** iPhone pronation/supination test configurations.

Test Type	Test Configuration
Analytical Validity	This test configuration was completed for both unsupervised and supervised completion of the task as instructed
Operational Tolerance	The test was completed for each of the following configurations: Completing the task as instructed while also raising and lowering the arm during the task; Stopping and starting the turning of the phone every 5 s; Stopping the assessment after 2 turns and placing the iPhone on a table while the test timed out; Completing the task as instructed while also rotating the phone about 3 axes, i.e., both intended and orthogonal motion.

**Table 6:** ActiGraph passive walking detection and gait test configurations.

Test	Test Configuration
Analytical Validity 10 s walk	Wear the ActiGraph on the dominant wrist; Stand still for 10 s; Walk for 10 s; Stop for 10 s; Repeat walk and stop for a total of 6 times; Turn around when necessary; Stand still for 10 s at end of test. Rater one instructed the participant when to stop and start walking and overall time keeping. Rater two noted the start and stop time of each 10 s walk and both raters two and three counted the steps for each walking period.
Analytical Validity 20 s walk	Wear the ActiGraph on the dominant wrist; Stand still for 10 s; Walk for 20 s; Stop for 20 s; Repeat walk and stop for a total of 4 times; Turn around when necessary; Stand still for 10 s at end of test. Rater one instructed the participant when to stop and start walking and overall time keeping. Rater two noted the start and stop time of each 20 s walk and both raters two and three counted the steps for each walking period.

**Table 7:** Percent of ICC for agreement > 0.75 (ICC rated good or excellent) between each of the iPhone and ActiGraph device accelerometers and the Shake Table.

Device	Nominal Peak Acceleration	Percent of ICC > 0.75
iPhone	0.005 g to 3.261 g	99.4%
ActiGraph	≥ 0.1 g	91.9%
	<0.1 g	2.3%

**Table 8:** iPhone walking test ICC results for agreement between algorithm and rater measures per test type, analytical validity (AV) or operational tolerance (OT), test configuration and measure.

Type	Test	Duration (s)	Distance (m)	Steps (Count)	Speed (ms^−1^)	Stride Period (s)
AV	5 s Walk	0.496 *	0.856	0.838	0.730	0.334
	10 s Walk	−0.112 *	0.948	0.873	0.942	0.893
	15 s Walk	0.299 *	0.933	0.932	0.950	0.892
	20 s Walk	−0.206 *	0.944	0.976	0.944	0.955
	5–20 s Combined	0.989	0.987	0.992	0.754	0.593
OT	Loose Pocket	0.133 *	0.926	0.874	0.840	0.889
	Shoulder Bag	0.041 *	0.914	0.889	0.892	0.844

* the duration of each test is fixed. As a result there is no variation in rater duration and therefore poor ICC versus rater gold standard is a consequence of study design.

**Table 9:** iPhone pronation/supination ICC results for agreement between algorithm and rater measures per test type, analytical validity (AV) or operational tolerance (OT), test configuration and measure.

Type	Test	Turns (Count)	Rotation Rate (Turns/s)
AV	Complete as instructed	0.642	0.935
OT	Raise and lower arm	0.971	0.975
	Stop and start turn every 5 s	0.995	0.990
	Turn 2 times then stop	1.000	0.732

**Table 10:** ActiGraph passive walk detection and rater-matched gait measure analytical validity results.

Test	Statistic	Start Time (s)	End Time (s)	Duration (s)	Steps (Count)	Stride Period (s)
Start and stop walking every 10 s	MAE	0.874	0.521	0.494	0.628	0.039
	RMSE	0.999	0.626	0.629	0.816	0.051
	MAPE	-*	5.20%	5.00%	3.40%	3.60%
Start and stop walking every 20 s	MAE	1.048	1.158	1.121	2.036	0.028
	RMSE	1.545	1.492	1.645	3.166	0.036
	MAPE	-*	5.80%	5.60%	5.40%	2.60%
Combined results for start and stop every 10 s and 20 s	MAE	0.943	0.776	0.745	1.191	0.033
	RMSE	1.246	1.061	1.149	2.100	0.042
	MAPE	-*	5.50%	5.20%	4.20%	3.10%

## Data Availability

Data may be available upon written request.
